# EPHA7 mutation as a predictive biomarker for immune checkpoint inhibitors in multiple cancers

**DOI:** 10.1186/s12916-020-01899-x

**Published:** 2021-02-02

**Authors:** Zhen Zhang, Hao-Xiang Wu, Wu-Hao Lin, Zi-Xian Wang, Lu-Ping Yang, Zhao-Lei Zeng, Hui-Yan Luo

**Affiliations:** 1 State Key Laboratory of Oncology in South China, Collaborative Innovation Center for Cancer Medicine, Sun Yat-sen University Cancer Center, Sun Yat-sen University, Guangzhou, 510060 People’s Republic of China; 2 Research Unit of Precision Diagnosis and Treatment for Gastrointestinal Cancer, Chinese Academy of Medical Sciences, Guangzhou, 510060 People’s Republic of China

**Keywords:** Biomarker, Eph receptors, EPHA7, Immune checkpoint inhibitor, Pan-cancer

## Abstract

**Background:**

A critical and challenging process in immunotherapy is to identify cancer patients who could benefit from immune checkpoint inhibitors (ICIs). Exploration of predictive biomarkers could help to maximize the clinical benefits. Eph receptors have been shown to play essential roles in tumor immunity. However, the association between EPH gene mutation and ICI response is lacking.

**Methods:**

Clinical data and whole-exome sequencing (WES) data from published studies were collected and consolidated as a discovery cohort to analyze the association between EPH gene mutation and efficacy of ICI therapy. Another independent cohort from Memorial Sloan Kettering Cancer Center (MSKCC) was adopted to validate our findings. The Cancer Genome Atlas (TCGA) cohort was used to perform anti-tumor immunity and pathway enrichment analysis.

**Results:**

Among fourteen EPH genes, EPHA7-mutant (EPHA7-MUT) was enriched in patients responding to ICI therapy (FDR adjusted *P* < 0.05). In the discovery cohort (*n* = 386), significant differences were detected between EPHA7-MUT and EPHA7-wildtype (EPHA7-WT) patients regarding objective response rate (ORR, 52.6% vs 29.1%, FDR adjusted *P* = 0.0357) and durable clinical benefit (DCB, 70.3% vs 42.7%, FDR adjusted *P* = 0.0200). In the validation cohort (*n* = 1144), significant overall survival advantage was observed in EPHA7-MUT patients (HR = 0.62 [95% confidence interval, 0.39 to 0.97], multivariable adjusted *P* = 0.0367), which was independent of tumor mutational burden (TMB) and copy number alteration (CNA). Notably, EPHA7-MUT patients without ICI therapy had significantly worse overall survival in TCGA cohort (HR = 1.33 [95% confidence interval, 1.06 to 1.67], multivariable adjusted *P* = 0.0139). Further gene set enrichment analysis revealed enhanced anti-tumor immunity in EPHA7-MUT tumor.

**Conclusions:**

EPHA7-MUT successfully predicted better clinical outcomes in ICI-treated patients across multiple cancer types, indicating that EPHA7-MUT could serve as a potential predictive biomarker for immune checkpoint inhibitors.

**Supplementary Information:**

The online version contains supplementary material available at 10.1186/s12916-020-01899-x.

## Background

Immune checkpoint inhibitors (ICIs), including monoclonal antibodies that target the programmed cell death protein (ligand) 1 [PD-(L)1] and cytotoxic T lymphocyte-associated antigen 4 (CTLA-4), have revolutionized treatments across multiple cancer types [1–3]. However, despite the impressive success of ICIs, durable clinical responses vary among patients [4]. Thus, predictive biomarkers of ICI response are needed to deliver precise medical treatment [5].

As of today, PD-L1 expression, high microsatellite instability (MSI-H), tumor mutation burden (TMB), copy number alteration (CNA), neoantigen load (NAL), tumor immune microenvironment (TIME), gene expression profiles (GEPs), and some specific gene mutations were found associated with ICI response [6–11]. Among them, only a few biomarkers have been clinically validated and even those validated ones still had their limitations [3, 10, 12]. For example, in the CheckMate 568 study, 44–50% of patients with high TMB or high PD-L1 expression did not respond to ICIs while nearly 12–15% of patients with low TMB or low PD-L1 expression achieved a partial or complete response [10]. Therefore, exploration of novel precise biomarkers is required to maximize the clinical benefits.

As the largest family of receptor tyrosine kinases (RTKs), the erythropoietin-producing hepatocellular carcinoma (Eph) receptors are involved in a wide range of physiological activities, especially tumorigenesis, tumor immunity, and tumor angiogenesis [13–15]. Tumor angiogenesis is associated with immunosuppression [16]. Recent clinical trials showed that combination therapy of anti-angiogenesis and ICIs achieved more favorable outcomes than monotherapy in different cancers [17–19]. With the ability to promote tumor angiogenesis, Eph receptors have potential impacts on the efficacy of immunotherapy. Moreover, Eph receptors play important roles in anti-tumor immunity. For example, Eph receptors are source of tumor-associated antigen (TAA), which could elicit selective anti-tumor immunity [20]. Also, Yang et al. demonstrated Eph receptor-mediated cell contact-dependent juxtacrine signaling could reduce T cell-mediated anti-tumor immunity by upregulating PD-L1 expression [21]. Eph receptors are closely associated with immune response, and EPH genes are frequently mutated in various cancers [14]. Accordingly, genetic status of Eph receptors has potential predictive values in immunotherapy. However, the association between the genomic alterations of Eph receptor-related genes and ICI response has not been revealed.

In this study, we performed a comprehensive analysis of the predictive function of mutations in Eph receptor-related genes. And we uncovered that mutated EPHA7 was predictive of better clinical outcomes in patients receiving ICI therapy and strongly associated with enhanced anti-tumor immunity across multiple cancer types.

## Methods

### Discovery cohort

**Fig. 1:**
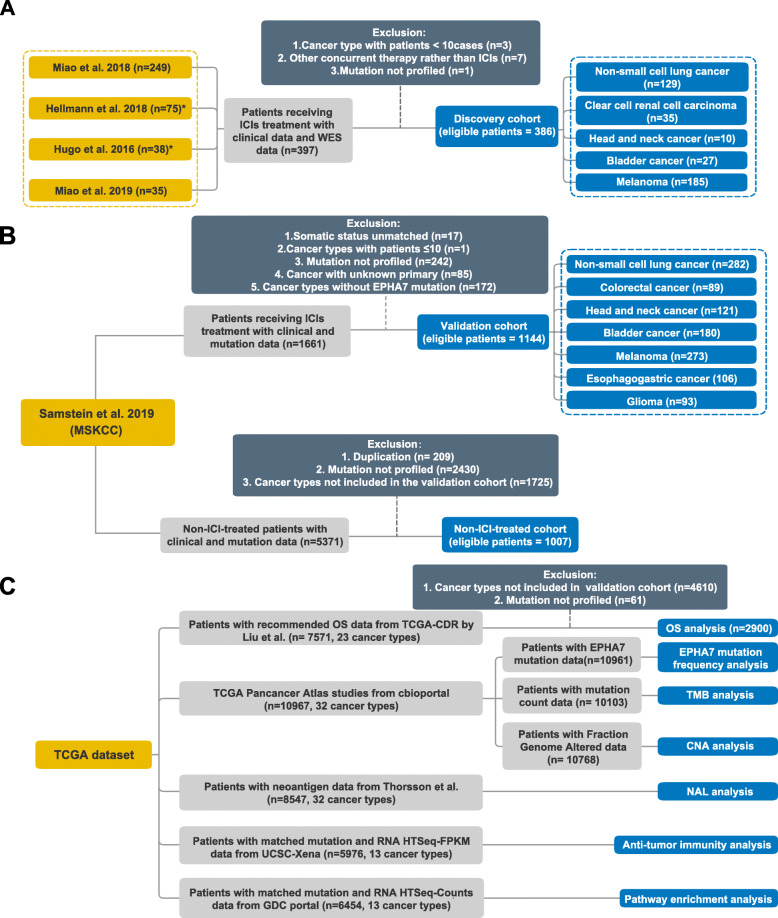
Flowchart of the study design. **a** Consolidation of the discovery cohort from seven published studies. Samples from the first four studies (Rizvi et al. [22], Snyder et al. [23], Van Allen et al. [24], Miao et al. [25]) have been curated and filtered by Miao et al. *Hellmann et al. cohort did not include OS data and Hugo et al. cohort did not include PFS data. **b** Consolidation of the validation cohort and the non-ICI-treated cohort from Samstein et al. **c** Consolidation of TCGA pan-cancer dataset. OS, overall survival; TMB, tumor mutation burden; CNA, copy number alteration; NAL, neoantigen analysis; GDC, Genomic Data Commons; MSKCC, Memorial Sloan Kettering Cancer Center

Eph receptors comprise 14 members, and each of them has a related gene (Additional file 1: Table S1). Some of these genes are not included in commercial targeted sequencing panels such as MSK-IMPACT. To evaluate the predictive functions of all these 14 genes in ICI-treated patients, we systematically collect annotated clinical data and whole-exome sequencing (WES) data from seven published studies on cBioPortal (https://www.cbioportal.org) (Fig. 1a) [22–28]. Samples from the first four studies have been curated and filtered by Miao et al. [25]. Totally, 386 patients from five cancer types were included in the discovery cohort.

### Validation cohort

Tumors with nonsynonymous somatic mutations in the coding region of EPHA7 were defined as EPHA7-mutant (EPHA7-MUT), while tumors without as EPHA7-wildtype (EPHA7-WT). To validate the predictive function of EPHA7 mutation, an independent pan-cancer cohort by Samstein et al. with only overall survival data and genomic data was retrieved from cBioPortal [29]. Samples from this cohort were sequenced using MSK-IMPACT panels, including 341-, 410-, and 468-gene panel. EPHA7 was not profiled in the 341-gene panel, and thus, samples tested by this panel were excluded. After filtering, 1144 patients from seven cancer types were included in the validation cohort (Fig. 1b). Also, the non-ICI-treated cohort from Samstein et al. was included to investigate whether the survival benefit in EPHA7-MUT patients was specific to ICI therapy.

### The Cancer Genome Atlas (TCGA) cohort

Survival data were retrieved from TCGA Pan-Cancer Clinical Data Resource (TCGA-CDR) determined by Liu et al., which was used to investigate the prognostic impact of EPHA7 mutation [30]. Somatic mutation data and fraction of altered genome data were retrieved from cBioPortal and neoantigen data was from Thorsson et al., which was used for the analysis of the association between EPHA7 mutation status and TMB, CNA, or NAL, respectively [31]. RNA-seq FPKM data were retrieved from UCSC Xena data portal (https://xenabrowser.net) for anti-tumor immunity analysis, and RNA-seq HTSeq-counts data was obtained from Genomic Data Commons (GDC) Data Portal (https://portal.gdc.cancer.gov/) for pathway enrichment analysis [32]. Processing and analyzing of TCGA data were shown in the flowchart (Fig. 1c).

### Clinical outcomes

The primary clinical outcomes were objective response rate (ORR), durable clinical benefit (DCB), progression-free survival (PFS), and overall survival (OS). ORR was assessed using Response Evaluation Criteria in Solid Tumors (RECIST) version 1.1 (irRECIST for the Hugo et al. study) [26]. DCB was defined as complete response (CR), partial response (PR), or stable disease (SD) lasting longer than 6 months; progression of disease (PD) or SD lasting less than 6 months was considered as no durable benefit (NDB). Patients who had not progressed and were censored before 6 months of follow-up were considered not evaluable (NE). PFS was assessed from the date the patient began immunotherapy to the date of progression or death of any cause. Patients who had not progressed were censored at the date of their last scan. Overall survival was calculated from the start date of ICI treatment in both discovery and validation cohorts, from the date of first infusional chemotherapy in the non-ICI-treated cohort, and from the date of first diagnosis in TCGA cohort, respectively.

### TMB and CNA data analysis

TMB was defined as the total number of nonsynonymous somatic, coding, base substitution, and indel mutations per megabase (Mb) of genome examined [33]. For WES data in the discovery cohort and TCGA cohort, 38 Mb was adopted as the estimated exome size [34]. For samples sequenced by MSK-IMPACT panel, the lengths of the captured region are 0.98, 1.06, and 1.22 Mb in 341, 410, and 468 gene panels, respectively. Mutations in driver oncogenes were not excluded from the validation cohort as described previously [29]. The cutoff value for high and low TMB in this study was the top 20% TMB within each cancer type [29].

Data of CNA in the validation cohort and TCGA cohort was obtained from cBioPortal and presented as the fraction of copy number altered genome. The cutoff value for high and low CNA in this study was the median CNA within each cancer type [6].

### Anti-tumor immunity and pathway enrichment analysis

To investigate the association between anti-tumor immunity and EPHA7 mutation, we evaluated tumor-infiltrating leukocytes and immune-related genes in TCGA cohort. Twenty-two immune cells’ infiltration status was analyzed using CIBERSORT web portal (https://cibersort.stanford.edu/) [35]. Immune-related genes and their functional classifications were obtained from Thorsson et al. [31].

To further characterize the TIME, we evaluated Hallmark pathways, Gene Ontology (GO), Kyoto Encyclopedia of Genes and Genomes (KEGG) pathways, and Reactome pathways in EPHA7-MUT and EPHA7-WT patients. R package DESeq2 was used for differential gene expression (DGE) analysis [36]. R package ClusterProfiler was used for gene set enrichment analysis (GSEA) [37].

### Statistical analysis

Statistical analyses were performed using R v. 4.0.2 (https://www.r-project.org). ORR and DCB in different subgroups based on specific gene status were analyzed by Fisher’s exact test, and the Benjamini-Hochberg procedure (B-H) was applied to control for false discovery rate (FDR). The Kaplan-Meier curve analysis of PFS and OS was compared using the log-rank test. The Cox proportional hazards model was applied for multivariate survival analysis, and available confounding factors were adjusted, including (1) age, sex, cancer type, drug class, and TMB level in the discovery cohort; (2) age, sex, cancer type, drug class, and TMB level in the validation cohort; (3) sex, cancer type, and TMB level in the non-ICI-treated cohort; and (4) age, sex, race, cancer type, histology grade, and tumor stage in TCGA cohort. Interactions between the EPHA7 status and the following factors were assessed in the validation cohort, including age, sex, cancer type, TMB level, and drug class. The differences of TMB, NAL, CNA, tumor-infiltrating leukocytes, and immune-related gene expressions between EPHA7-MUT and EPHA7-WT tumors were examined using the Mann-Whitney *U* test. All reported *P* values were two-tailed, and *P* < 0.05 was considered statistically significant.

## Results

### EPHA7-MUT predicted favorable clinical outcomes to ICIs in the discovery cohort

**Fig. 2:**
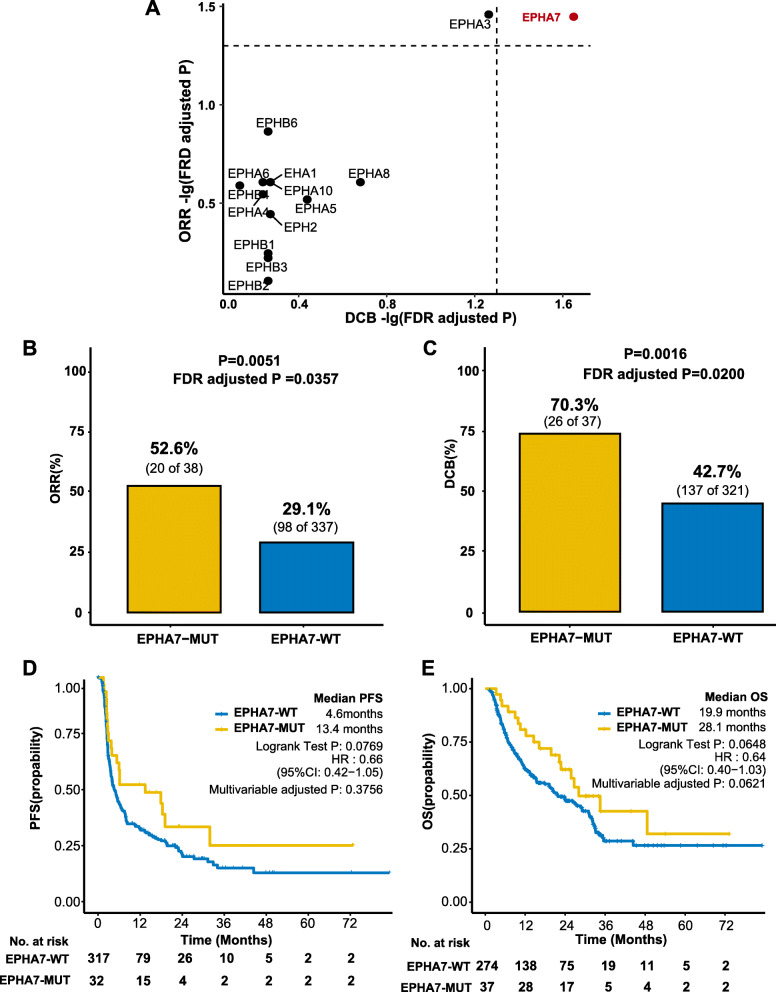
Association between EPH7A mutation and clinical outcomes in the discovery cohort. **a** Associations between EPH gene mutation and clinical responses (ORR and DCB). Both dashed lines indicated B-H adjusted *P* = 0.05 regarding DCB and ORR, respectively (two-tailed Fisher’s exact test). **b** Histogram depicting proportions of ORR in EPHA7-MUT and EPHA7-WT patients (two-tailed Fisher’s exact test). **c** Histogram depicting proportions of DCB in EPHA7-MUT and EPHA7-WT patients (two-tailed Fisher’s exact test). **d** The Kaplan-Meier survival analysis comparing PFS between EPHA7-MUT and EPHA7-WT patients in the discovery cohort (*n* = 349). There were 349 patients with available PFS data for PFS analysis. Missing PFS data consisted of 37 patients from Hugo et al. cohort. **e** The Kaplan-Meier survival analysis comparing OS between EPHA7-MUT and EPHA7-WT patients in the discovery cohort. There were 311 patients with available OS data for OS analysis. Missing OS data consisted of 75 patients from Hellman et al. cohort. HR and adjusted *P* in **d** and **e** were calculated by the Cox proportional hazards regression analysis. Available confounding factors were adjusted: age, sex, cancer type, drug class, and TMB level. ORR, objective response rate; SD, stable disease; PD, progressive disease; CR, complete response; PR, partial response; DCB, durable clinical benefit; NCB, no clinical benefits; PFS, progression-free survival; OS, overall survival; B-H: Benjamini-Hochberg procedure

The baseline patient characteristics of the discovery cohort were summarized in Table 1. Five cancer types were included: non-small cell lung cancer (NSCLC) (*n* = 129), melanoma (*n* = 185), clear cell renal cell carcinoma (*n* = 35), bladder cancer (*n* = 27), and head and neck cancer (*n* = 10). Fourteen Eph receptor-related genes, including EPHA1, EPHA2, EPHA3, EPHA4, EPHA5, EPHA6, EPHA7, EPHA8, EPHA10, EPHB1, EPHB2, EPHB3, EPHB4, and EPHB6, were investigated. Among these 14 genes, EPHA7-MUT was the only one that significantly gathered in patients with both ORR and DCB (Fig. 2a, both adjusted *P* < 0.05). This indicated that EPHA7-MUT may potentially predict the efficacy of ICI treatment.

**Table 1:** Patient characteristics in the discovery cohort

Characteristics	No. (%)
**Gender**	
Male	234 (60.6)
Female	152 (39.4)
**Age**	
≥ 60	157 (40.7)
< 60	229 (59.3)
**Cancer type**	
Non-small cell lung cancer	129 (33.4)
Melanoma	185 (47.9)
Clear cell renal cell carcinoma	35 (9.1)
Bladder cancer	27 (7.0)
Head and neck cancer	10 (2.6)
**Drug class**	
CTLA-4 (mono)	142 (36.8)
PD-(L)1 (mono)	115 (29.8)
CTLA-4 + PD-(L)1 (combo)	129 (33.4)
**Best overall response**	
CR/PR	118 (30.6)
SD	94 (24.4)
PD	163 (42.2)
NE^a^	11 (2.8)
**Durable clinical benefit**	
DCB	163 (42.2)
NDB	195 (50.5)
NE^b^	28 (7.3)
**EPHA7 status**	
EPHA7-WT	348 (90.2)
EPHA7-MUT	38 (9.8)
**Overall patients**	386

*Abbreviations*: *CR* complete response, *CTLA-4* cytotoxic T cell lymphocyte-4, *DCB* durable clinical benefit, *NDB* no durable benefit, *NE* not evaluable, *PD* progressive disease, *PD-(L)1* programmed cell death-1 or programmed death-ligand 1, *PR* partial response, *SD* stable disease

^a^Eleven patients with best overall response not evaluable due to missing data, including four from Miao et al. [25] and seven from Hellmann et al. [27]

^b^Twenty-eight patients with durable clinical benefit not evaluable, including 11 missing data and 17 patients who had not progressed but were censored before 6 months of follow-up

Patients’ characteristics stratified by EPHA7 status in the discovery cohort were shown in Additional file 2: Table S2. There were 38 EPHA7-MUT patients, including 33 melanomas (3 CR, 13 PR, 7 SD, and 9PD), 2 non-small cell lung cancers (2 PR), 2 clear cell renal cell carcinomas (1 SD and 1 PR), and 1 bladder cancer (1SD). Detailed analysis of ORR, DCB, PFS, and OS between EPHA7-MUT and EPHA7-WT was presented in Fig. 2b–e. The proportion of CR/PR in EPHA7-MUT patients was almost as twice as that in EPHA7-WT patients (52.6% vs 29.1%, *P* = 0.0051, FDR adjusted *P* = 0.0357). Proportion of DCB in EPHA7-MUT patients was 27.6% higher than that in EPHA7-WT patients (70.3% vs 42.7%, *P* = 0.0016, FDR adjusted *P* = 0.0200). Longer PFS was detected in EPHA7-MUT patients (median PFS 13.4 months vs 4.6 months, hazard ratio [HR] = 0.66 [95% CI, 0.42–1.05], log-rank test *P* = 0.0769, multivariable adjusted *P* = 0.3756). As for OS analysis, median OS was 28.1 months in EPHA7-MUT patients, which was 8.2 months longer than in EPHA7-WT patients (HR = 0.64 [95% CI, 0.40–1.03], log-rank test *P* = 0.0648, multivariable adjusted *P* = 0.0621). After adjusted for sex, age, cancer types, drug class, and TMB level, numerical OS benefit still existed. However, significant difference of PFS and OS was not observed, probably due to limited sample size.

### EPHA7-MUT predicted survival advantage in the validation cohort

**Fig. 3:**
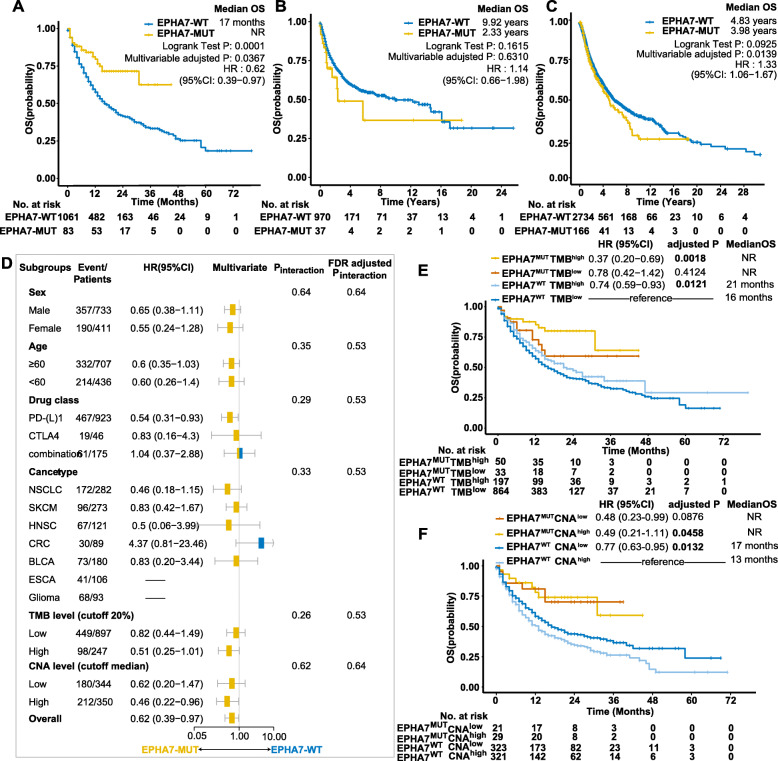
Validation of the predictive value of EPHA7-MUT. **a** The Kaplan-Meier curves comparing OS between EPHA7-MUT and EPHA7-WT patients in the validation cohort. **b** The Kaplan-Meier curves comparing OS between EPHA7-MUT and EPHA7-WT patients in the non-ICI-treated cohort. **c** The Kaplan-Meier curves comparing OS between EPHA7-MUT and EPHA7-WT patients in TCGA cohort. **d** Forest plot depicting subgroup analysis in the validation cohort. Drug class “Combination” indicated combination therapy of CTLA-4 and PD-(L)1 antibodies. EPHA7-MUT cases were insufficient for hazard ratio calculation in ESCA and glioma subgroups. There were only 694 patients with available CNA data for survival analysis. NSCLC, non-small cell lung cancer; SKCM, melanoma; HNSC, head and neck cancer; CRC, colorectal cancer; BLCA, bladder cancer; ESCA, esophagogastric cancer. **e** The Kaplan-Meier curves comparing OS among EPHA7^MUT^TMB^high^, EPHA7^MUT^TMB^low^, EPHA7^WT^TMB^high^, and EPHA7^WT^TMB^low^ groups in the validation cohort. **f** The Kaplan-Meier curves comparing OS among EPHA7^MUT^CNA^high^, EPHA7^MUT^CNA^low^, EPHA7^WT^CNA^high^, and EPHA7^WT^CNA^low^ groups in the validation cohort. HR and adjusted *P* were calculated by the Cox proportional hazards regression analysis. Available confounding factors were adjusted: validation cohort (age, sex, cancer type, drug class, TMB level), non-ICI-treated cohort (sex, cancer type, TMB level), and TCGA cohort (age, sex, race, cancer type, histology grade, tumor stage). NR indicated the median OS has not been reached

To further investigate the survival benefit in ICI-treated patients with EPHA7 mutation, we performed the survival analysis in an independent validation cohort with a larger sample size (*n* = 1144). There were 83 EPHA7-MUT patients including 45 melanomas, 18 non-small cell lung cancers, 5 head and neck cancer cell carcinomas, 5 bladder cancers, 5 colorectal cancers, 4 esophagogastric cancers, and 1 glioma, which took up 7.3% of the population in the validation cohort. After adjusting confounding factors (sex, age, cancer type, drug class, and TMB level), EPHA7-MUT patients achieved significantly longer OS than EPHA7-WT patients in the validation cohort (median OS: not reach [NR] vs 17 months, HR = 0.62 [95% CI, 0.39–0.97], log-rank test *P* = 0.0001, multivariable adjusted *P* = 0.0367) (Fig. 3a). In the non-ICI-treated cohort, there were no significant differences between EPHA7-MUT and EPHA7-WT patients (median OS 2.33 years [MUT] vs 9.92 years [WT], HR = 1.14 [95% CI, 0.66–1.98], log-rank test *P* = 0.1615, multivariable adjusted *P* = 0.6310) (Fig. 3b). In TCGA cohort, however, significantly worse overall survival was observed in EPHA7-MUT patients (median OS 3.98 years [MUT] vs 4.83 years [WT], HR = 1.33 [95% CI, 1.06–1.67], log-rank test *P* = 0.0925, multivariable adjusted *P* = 0.0139) (Fig. 3b, c).

In subgroup analysis, the survival advantage of EPHA7-MUT vs EPHA7-WT was prominent and consistent across sex, age, drug class, cancer type (except for colorectal cancer), TMB level, and CNA level (Fig. 3d, all *P*_interaction_ > 0.05). Interestingly, colorectal cancer patients achieved longer survival with EPHA7-WT instead of EPHA7-MUT (HR = 4.37 [95% CI 0.81–23.46], adjusted *P* = 0.08). EPHA7-MUT patients presented with higher TMB (*P* < 0.0001) and CNA (*P* = 0.0126) in the validation cohort (Additional file 3: Figure S1). According to EPHA7 status and TMB level, we divided patients into four groups: EPHA7^MUT^TMB^high^, EPHA7^MUT^TMB^low^, EPHA7^WT^TMB^high^, and EPHA7^WT^TMB^low^. As expected, EPHA7^MUT^TMB^high^ patients achieved the longest OS among all groups (Fig. 3e). In high-TMB patients, EPHA7-MUT successfully identified patients with better survival benefit (HR = 0.49 [95% CI 0.26–0.95], adjusted *P* = 0.035). The same analysis was applied to CNA as well (Fig. 3f). There were 694 patients with available CNA data in the validation cohort. Notably, even in high-CNA patients, EPHA7-MUT still managed to predict a better survival (HR = 0.49 [95% CI 0.21–1.11], adjusted *P* = 0.0458).

EPHA7-MUT patients were further stratified into truncating EPHA7-MUT and non-truncating EPHA7-MUT subgroups in both discovery and validation cohorts. There are no significant differences between these two groups, which was presented in Additional file 4: Figure S2.

### Mutation frequency, anti-tumor immunity, and pathway enrichment analysis of EPHA7-MUT in TCGA cohort

**Fig. 4:**
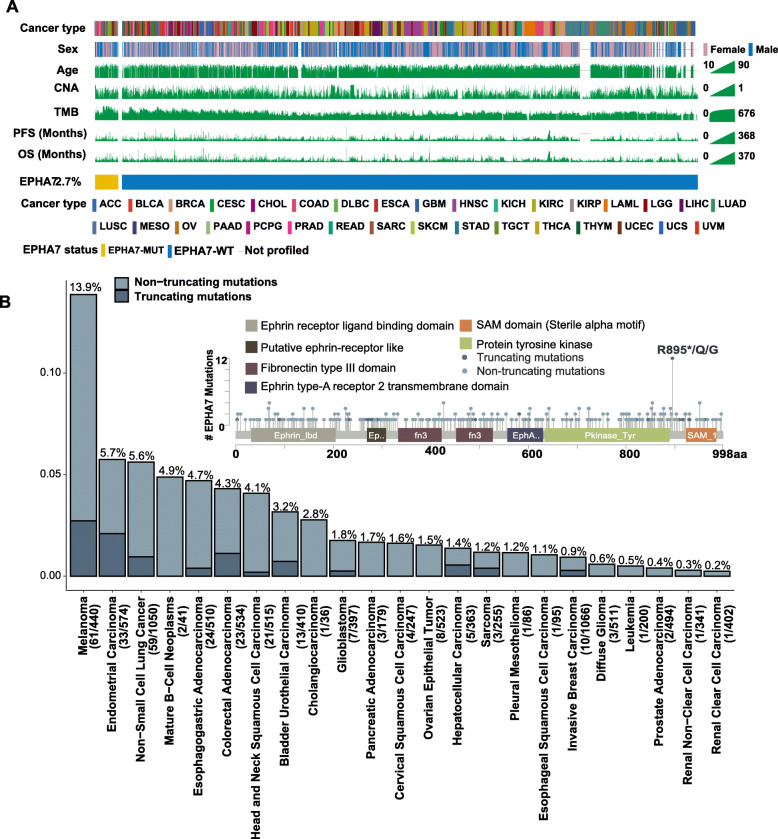
Mutational landscape of EPHA7 in TCGA cohort. **a** Association of EPHA7 status and clinical characteristics in TCGA cohort. The cancer type, sex, age, CNA, TMB, PFS, and OS were annotated. Samples were sorted by EPHA7 status, while EPHA7-MUT and EPHA7-WT samples were separated by a gap. **b** The proportion of EPHA7-MUT tumors identified in each cancer type with at least one mutation case. Numbers above the barplot indicated the alteration frequency, and numbers close to cancer names indicated the number of EPHA7-MUT patients and the total number of patients. “Truncating mutations” included nonsense, splice site mutations, and frameshift insertion and deletion; “Non-truncating mutations” included missense mutations and inframe insertion and deletion

Mutational landscape of EPHA7 and its association with clinical characteristics were shown in Fig. 4a. The overall mutation frequency of EPHA7 was 2.7% (287/10,437) in TCGA pan-cancer cohort with melanoma (13.6%) ranking first followed by non-small cell lung cancer (5.6%) and endometrial carcinoma (5.6%) (Fig. 4b). The most frequent somatic mutation site of EPHA7 was p.R895, and generally, somatic mutations were evenly distributed without any annotated functional hotspot mutations from 3D Hotspots (https://www.3dhotspots.org) [38].

**Fig. 5:**
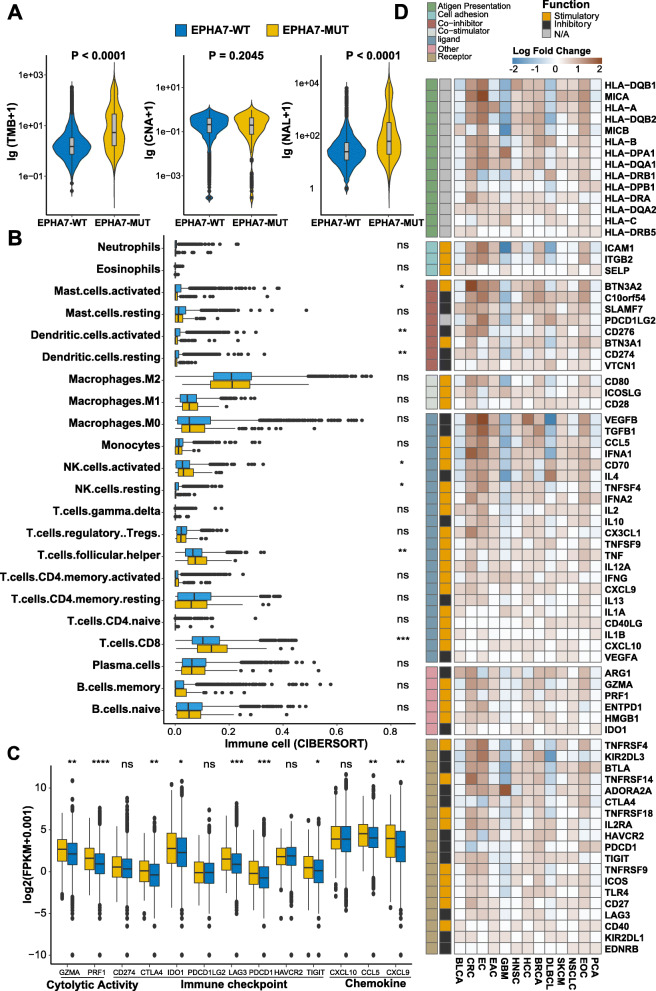
EPHA7-MUT was associated with enhanced anti-tumor immunity in TCGA cohort. **a** Violin plot depicting the distribution of TMB, CNA, and NAL in EPHA7-MUT and EPHA7-WT tumors. **b** Boxplot depicting the infiltration of 22 immune cells in EPHA7-MUT and EPHA7-WT tumors. CIBERSORT was used to calculate the infiltration degree of these immune cells. Gene expression profiles were uploaded to CIBERSORT web portal, and the algorithm was configured with 1000 permutations. CIBERSORT results were recorded in Additional file 5: Table S3. Samples with deconvolution *P* value ≥ 0.05 were excluded (*n* = 2967) (Mann-Whitney *U* test; ns, not significant; *P < 0.05, **P < 0.01, ***P < 0.001, ****P < 0.0001). **c** Boxplot depicting the expression level of immune-related genes in EPHA7-MUT and EPHA7-WT groups (Mann-Whitney *U* test; ns, not significant; *P < 0.05, **P < 0.01, ***P < 0.001, ****P < 0.0001). **d** Heatmap depicting the log2-transformed fold change in the expression level of immune-related genes across multiple cancer types (EPHA7-MUT vs EPHA7-WT). Blue indicated downregulation and red indicated upregulation

EPHA7-MUT was associated with increased immunogenicity. TMB and NAL were higher in EPHA7-MUT tumors (both *P* < 0.0001), while CNA remained similar in both EPHA7-MUT and EPHA7-WT tumors (*P* = 0.2045) (Fig. 5a). Also, we used CIBERSORT to investigate infiltration of immune cells and results were recorded in Additional file 5: Table S3. As expected, enhanced anti-tumor immunity was observed in EPHA7-MUT tumors. Cytotoxic lymphocytes, including activated NK cells (*P* < 0.05) and cytotoxic T cells (*P* < 0.001), were more abundant in EPHA7-MUT tumors (Fig. 5b). Expression of cytotoxic activity-related genes (GZMA, PRF1), chemokine-related genes (CCL5, CXCL9), and checkpoint-related genes (PDCD1, LAG3, IDO1, CTLA-4, TIGHT) were also upregulated in EPHA7-MUT tumors (Fig. 5c, all *P* < 0.01). To further investigate the association between anti-tumor immunity and EPHA7-MUT across multiple cancer types, we thoroughly examined immune-related genes within each cancer type. A general upregulation of stimulatory immunomodulators was observed in EPHA7-MUT tumors except glioblastoma (GBM), which showed a general downregulation of both inhibitory and stimulatory immunomodulators (Fig. 5d).

**Fig. 6:**
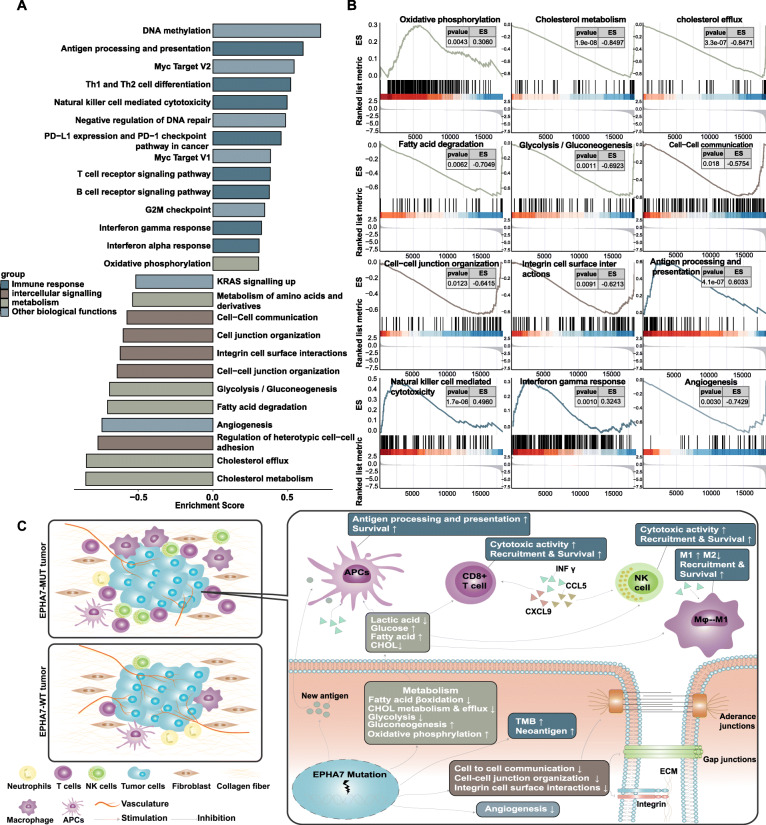
Pathway enrichment analysis in TCGA dataset and possible tumor immune microenvironment in EPHA7-MUT and EPHA7-WT tumors. **a** Differences in pathway activities scored by GSEA between EPHA7-MUT and EPHA7-WT tumors in TCGA dataset. Significant results (*P* < 0.05 and FDR < 0.25) of enrichment analysis were summarized in Additional file 6: Table S4. Pathways which might potentially impact the tumor immune microenvironment were presented in **a**. These pathways were divided into four groups: immune function (blue), intercellular signaling (brown), metabolism (green), and other biological functions (gray). **b** GSEA plot depicting representative pathways identified by GSEA between EPHA-MUT and EPHA7-WT tumors, including metabolism, cell communication, immune response, and angiogenesis. **c** Comparison of possible tumor immune microenvironment between EPHA7-MUT and EPHA7-WT tumors. APCs, antigen presenting cells; NK cell, nature killer cell; ECM, extracellular matrix

The results of enrichment analysis showed that several pathways varied significantly between EPHA7-MUT and EPHA7-WT tumors, including metabolism, intercellular interaction, immune function, and other biological functions (Fig. 6a). Significant results (*P* < 0.05 and FDR < 0.25) of enrichment analysis were summarized in Additional file 6: Table S4. Cholesterol efflux and metabolism, fatty acid degradation, glycolysis, cell-cell communication, cell-cell junction organization, integrin cell surface interactions, and angiogenesis were downregulated in EPHA7-MUT tumors (Fig. 6b, all *P* < 0.05). Oxidative phosphorylation, antigen processing and presentation, NK-mediated cytotoxicity, and interferon gamma response were upregulated in EPHA7-MUT tumors (Fig. 6b, all *P* < 0.05). According to the results of pathway enrichment analysis, the possible TIME of EPHA7-MUT and EPHA7-WT tumor was summarized in Fig. 6c.

## Discussion

In our study, we systematically collected and consolidated both clinical and genomic data to evaluate the association between EPH gene status and clinical responses in ICI-treated cancer patients. Then, we carefully validated our findings in another independent cohort and thoroughly explored the corresponding TIME. We found EPHA7-MUT was significantly associated with better clinical outcomes in ICI-treated patients and enhanced anti-tumor immunity. Remarkably, this predicting value of EPHA7-MUT was independent of TMB and CNA. This is the first study performing a comprehensive analysis of the relationship between EPH gene mutation status and clinical outcomes in ICI-treated patients across multiple cancer types.

We found some meaningful changes in biological functions of EPHA7-MUT tumors, including intercellular communication, angiogenesis, and metabolism. First of all, Eph receptors and their ligands (ephrin) have been proven essential in the cell communication system [14]. Hence, it is reasonable to observe a downregulation of intercellular communication in EPHA7-MUT tumors in our analysis. Previous study showed that inhibiting intercellular communication by targeting EPHA10 could boost anti-tumor immunity by reducing PD-L1 expression [21]. However, we found an upregulation of PD-L1 expression in EPHA7-MUT tumors with decreased cell-cell contact. This finding suggested that decreased intercellular communication in EPHA7-MUT tumors may have other underlying mechanisms that enhance anti-tumor immunity rather than depressing PD-L1 expression. Secondly, Eph-ephrin signaling promotes tumor angiogenesis [14]. As expected, we found the angiogenesis pathway was significantly downregulated in EPHA7-MUT tumors. Angiogenesis and immunosuppression are closely related. Tortuous tumor vasculature causes a hypoxic tumor environment and hinders the infiltration of lymphocytes [39]. Decreased angiogenesis in EPHA7-MUT tumors could promote lymphocyte infiltration. Finally, metabolic changes in EPHA7-MUT tumors could also strengthen anti-tumor immunity. Fatty acids and glucose promote the survival of immune cells in the tumor microenvironment, while cholesterol and lactate function reversely [40]. Accumulation of fatty acids and glucose, and depletion of cholesterol and lactate within EPHA7-MUT tumor were detected in our analysis, which could create a better TIME and enable cytotoxic lymphocytes to work more effectively. Overall, EPHA7-MUT tumors are more likely to provide a friendly living environment for those effective immune cells and thus enhance the anti-tumor immunity.

Ephrin receptors form a large family of receptor tyrosine kinase and regulate various biological functions. Both oncogenic and tumor suppressive roles have been reported for specific ephrin receptors [14]. Particularly, EPHA7 has been previously identified as a tumor suppressive gene that inhibits tumor growth and progression in various cancers [41, 42]. Survival analysis in both non-ICI-treated cohort and TCGA cohort revealed longer median OS in EPHAT-WT instead of EPHA7-MUT patients, indicating that EPHA7-MUT might potentially have a worse prognostic impact on cancer patients. Accordingly, the clinical benefits of EPHA7-MUT patients with ICIs should be the result that the ICI treatment benefits of EPHA7-MUT outweighed its harmful prognostic impact. We then further analyzed cancer subgroups in TCGA cohort, which showed a generally and numerically worse prognosis of EPHA7-MUT patients within each cancer type (Additional file 7: Figure S3). Colorectal cancer with EPHA7-MUT had the worst prognosis in TCGA cohort (HR = 5.21 [95% CI, 2.22–12.21], adjusted *P* < 0.0001). This could partially explain why EPHA7-MUT colorectal cancer was the only one that presented with worse OS in the validation cohort, because the harmful prognostic impact of EPHA7-MUT outweighed its ICI treatment benefits in colorectal cancer. Also, besides the aforementioned sample size of the discovery cohort, this assumption could be another reason why only numerical survival advantage of EPHA7-MUT patients was observed in the discovery cohort, since the harmful prognostic impact of EPHA7-MUT could partly mask the ICI therapy benefits. Notably, these findings supported our previous assumption and further stressed the importance of ICI therapy in EPHA7-MUT patients, which could potentially turn the harmful prognostic impact of EPHA7-MUT patients into an overall survival benefit.

In the initial screening process, EPHA3 was ruled out since its FDR adjusted *P* value of DCB was 0.053. However, given this borderline *P* value, EPHA3 was worth following up. Hence, we have also done the survival analysis of EPHA3 in both discovery cohort and validation cohort. Results could be found in the supplementary material (Additional file 8: Figure S4). However, only numerical survival benefits were observed in both discovery and validation cohort for EPHA3-MUT patients. Therefore, EPHA3 might not be as effective as EPHA7 in predicting the efficacy of immunotherapy in current analysis.

In our primary analysis, it is individual EPH gene that was evaluated rather than cumulative effects of all 14 EPH genes. To investigate the combined effects of all 14 EPH genes, we performed further analysis combining all 14 genes (Additional file 9: Figure S5). EPH-MUT was defined as at least one EPH gene has mutation among 14 genes, while EPH-WT was defined as none of EPH genes has mutation. Although EPH-MUT patients presented with a higher ORR than EPH-WT patients (40.8% vs 24.8%), there were no significant differences between EPH-MUT and EPH-WT patients regarding DCB, PFS, and OS. Activation of different Eph receptors can have highly varied impacts on cellular processes, but exact function of each Eph receptor has not been fully understood. Therefore, it would be more reasonable to test the combined predictive value of EPH genes that have synergic effect in the future rather than all EPH genes.

This retrospective analysis also has several limitations. Firstly, only four out of fourteen EPH genes are included in the MSK-IMPACT panel. To analyze all EPH genes, we only included cohorts with WES data in the discovery cohort. Considering the limited sample size of the discovery cohort (*n* = 386), we should not completely exclude the predictive function of other EPH genes. Secondly, mutation rate of EPHA7 in melanoma was nearly 2.5 times higher than in other cancer types. The majority of EPHA-MUT samples were melanoma (33/38) in the discovery cohort, which is a major confounding factor causing bias. However, the survival advantages across multiple cancers in the validation cohort as well as the general upregulation of anti-tumor immunity in various cancers could compensate the bias to some degree. Still, the predictive value of EPHA7 mutation with regard to cancer types needs to be verified in future prospective trials. Additionally, the possible TIME and molecular mechanisms of EPHA7-MUT were demonstrated based on GSEA, which requires further molecular researches to validate. Finally, gene expression data has not been included in both the discovery and validation cohorts. Therefore, combination analysis of EPHA7 and other predictive biomarkers (e.g., expression of PD-L1) has not been performed. Clinical trials with expression data are needed to expand our findings and test the added value of tumor-infiltrating lymphocytes in the survival analysis of EPHA7-MUT.

Importantly, these limitations do not preclude the favorable clinical outcomes derived from immunotherapy in EPHA7-MUT patients. Unlike continuous variables such as TMB, CNA, or PD-L1 expression, EPHA7-MUT are easily detected by NGS and clearly classify patients into two groups that are associated with immunotherapy response. The scope of EPHA7-MUT falls in compensating the existing biomarkers to detect those patients who are most likely to benefit from immunotherapy. Our study not only paved the way for precise treatments tailored to molecular subtypes, but also indicated the association between Eph receptor-related TIME and immunotherapy response. Biological functions mediated by Eph receptors, especially tumor angiogenesis, intercellular contact, and tumor metabolism, should be better characterized in future studies. Although there are some researches or ongoing trials co-targeting these pathways and tumor immunity [18, 40], our study introduces a novel angle that Eph receptors might influence the immunotherapy response through corresponding biological functions. Further elucidation of the molecular mechanism between Eph receptors and anti-tumor immunity is warranted to validate the predictive value as well as help to identify potential therapeutic targets.

## Conclusion

Our study demonstrated the robust link between EPHA7-MUT and better clinical outcomes in ICI-treated cancer patients. Therefore, EPHA7-MUT has the potential to serve as a predictive biomarker for immune checkpoint blockades across multiple cancer types. Validation of the predictive value in future prospective trials and exploration of the molecular mechanism in further molecular researches are warranted for EPHA7-MUT.

## Supplementary Information

**Additional file 1: Table S1.** EPH genes and corresponding clinical outcomes in the discovery cohort.

**Additional file 2: Table S2.** Patient characteristics in the discovery cohort stratified by EPHA7 status.

**Additional file 3: Figure S1.** Violin plot depicting the distribution of TMB and CNA in EPHA7-MUT and EPHA7-WT tumors.

**Additional file 4: Figure S2.** Truncating vs non-truncating EPHA7 mutation analysis in both discovery and validation cohort. Figure S2 A-D: discovery cohort. Figure S2 E: validation cohort.

**Additional file 5: Table S3.** Results of CIBERSORT analysis in TCGA cohort.

**Additional file 6: Table S4.** Significant pathways detected by gene set enrichment analysis. (EPHA7-MUT vs EPHA7-WT tumors).

**Additional file 7: Figure S3.** Survival analysis of cancer subgroups in the TCGA cohort.

**Additional file 8: Figure S4.** Survival analysis of EPHA3 in both discovery and validation cohort.

**Additional file 9: Figure S5.** Association between clinical outcomes and the combination of all 14 EPH genes in the discovery cohort.

## Data Availability

All of the data we used in this study were publicly available as described in the “Methods” section.

## Abbreviations

ACC: Adrenocortical carcinoma
BLCA: Bladder urothelial carcinoma
BRCA: Invasive breast carcinoma
CHOL: Cholangiocarcinoma
CI: Confidence interval
CNA: Copy number alteration
COAD: Colorectal adenocarcinoma
CR: Complete response
CRC: Colorectal cancer
CSCC: Cervical squamous cell carcinoma
CTLA-4: Cytotoxic T lymphocyte antigen 4
DCB: Durable clinical benefit
DLBC: Diffuse large B cell lymphoma
EAC: Esophagogastric adenocarcinoma
EC: Endometrial carcinoma
ECA: Endocervical adenocarcinoma
ESCC: Esophageal squamous cell carcinoma
FDA: Food and Drug Administration
FDR: False discovery rate
FPKM: Fragments per kilobase of exon model per million mapped fragments
GBM: Glioblastoma
HCC: Hepatocellular carcinoma
HNSC: Head and neck squamous cell carcinoma
HR: Hazard ratio
ICIs: Immune checkpoint inhibitors
KIRC: Kidney renal clear cell carcinoma
KIRP: Kidney renal papillary cell carcinoma
LAML: Acute myeloid leukemia
LGG: Diffuse glioma
Mb: Megabase
MESO: Mesothelioma
MSI-H: High microsatellite instability
NAL: Neoantigen load
NSCLC: Non-small cell lung cancer
ORR: Objective response rate
OS: Overall survival
OV: Ovarian epithelial tumor
PAAD: Pancreatic adenocarcinoma
PCC: Pheochromocytoma
PD: Progression of disease
PD-(L)1: Programmed cell death (ligand) 1
PFS: Progression-free survival
PGL: Paraganglioma
PRAD: Prostate adenocarcinoma
PR: Partial response
RECIST: Response Evaluation Criteria in Solid Tumors
SARC: Sarcoma
SD: Stable disease
SKCM: Melanoma
STAD: Stomach adenocarcinoma
TCGA: The Cancer Genome Atlas
EPHA7-MUT: EPHA7-mutant
EPHA7-WT: EPHA7-wildtype
TMB: Tumor mutational burden
UCES: Uterine corpus endometrial carcinoma
UVM: Uveal melanoma
WES: Whole-exome sequencing
