# Supplementary material for: EPHA7 mutation as a predictive biomarker for immune checkpoint inhibitors in multiple cancers
Source: BMC Med. 2021 Feb 2;19:26. doi: 10.1186/s12916-020-01899-x (PMC7852135; doi:10.1186/s12916-020-01899-x)
Supplement: Supplementary file 1 — Additional file 1: Table S1. EPH genes and corresponding clinical outcomes in the discovery cohort. [file 12916_2020_1899_MOESM1_ESM.docx]

| **Table S1 EPH genes and corresponding clinical outcomes in the discovery cohort** | | | | | | | | |
| --- | --- | --- | --- | --- | --- | --- | --- | --- |
| Gene Symbol | Gene name | Gene location | MSK-IMPACT panel^#^ | Cases with gene mutations | Total cases | Proportion | ORR  P value^1^  (FDR adjusted) | DCB  P value^2^  (FDR adjusted) |
| EPHA1 | Ephrin receptor A1 | 7q33-35 | not included | 13 | 386 | 3.37% | 0.247 | 0.535 |
| EPHA2 | Ephrin receptor A2 | 1p36 | not included | 16 | 386 | 4.15% | 0.359 | 0.535 |
| EPHA3 | Ephrin receptor A3 | 3p11.2 | Included | 35 | 386 | 9.07% | 0.036 | 0.053 |
| EPHA4 | Ephrin receptor A4 | 2q36.1 | not included | 26 | 386 | 6.74% | 0.284 | 0.579 |
| EPHA5 | Ephrin receptor A5 | 4q13.1 | Included | 21 | 386 | 5.44% | 0.303 | 0.364 |
| EPHA6 | Ephrin receptor A6 | 3q11.2 | not included | 42 | 386 | 10.88% | 0.247 | 0.579 |
| EPHA7 | Ephrin receptor A7 | 6q16.1 | included | 38 | 386 | 9.84% | 0.036 | 0.020 |
| EPHA8 | Ephrin receptor A8 | 1p36 | not included | 18 | 386 | 4.66% | 0.247 | 0.210 |
| EPHA10 | Ephrin receptor A10 | 1p34.3 | not included | 13 | 386 | 3.37% | 0.247 | 0.535 |
| EPHB1 | Ephrin receptor B1 | 3q22.2 | included | 23 | 386 | 5.96% | 0.569 | 0.548 |
| EPHB2 | Ephrin receptor B2 | 1p36 | not included | 16 | 386 | 4.15% | 0.784 | 0.548 |
| EPHB3 | Ephrin receptor B3 | 3q21 | not included | 13 | 386 | 3.37% | 0.600 | 0.548 |
| EPHB4 | Ephrin receptor B4 | 7q22 | not included | 9 | 386 | 2.33% | 0.257 | 0.737 |
| EPHB6 | Ephrin receptor B6 | 7q34 | not included | 27 | 386 | 6.99% | 0.137 | 0.548 |
| ^#^ Indicated that whether the genes were included in the MSK-IMPACT panel. EPHA7 was not included in the 341-gene panel. | | | | | | | | |
| ^1^P value < 0.05 denoted significantly enrichment in patients responding to immune check points inhibitor. | | | | | | | | |
| ^2^P value < 0.05 denoted significantly enrichment in patients who had durable clinical benefit with immune check points inhibitor. | | | | | | | | |
| Abbreviation: ORR, objective response rate; DCB, durable clinical benefit; FDR, false discovery rate. | | | | | | | | |
