# Supplementary material for: EPHA7 mutation as a predictive biomarker for immune checkpoint inhibitors in multiple cancers
Source: BMC Med. 2021 Feb 2;19:26. doi: 10.1186/s12916-020-01899-x (PMC7852135; doi:10.1186/s12916-020-01899-x)
Supplement: Supplementary file 2 — Additional file 2: Table S2. Patient characteristics in the discovery cohort stratified by EPHA7 status. [file 12916_2020_1899_MOESM2_ESM.docx]

| **Table S2.** Patient characteristics in the discovery cohort stratified by EPHA7 status | | | | |
| --- | --- | --- | --- | --- |
| **Characteristic** | **No. (%)** | **EPHA7 status [No. (%)^a^]** | | |
|  |  | **EPHA7-MUT** | | **EPHA7-WT** |
| **Gender** |  |  |  | |
| Male | 234 (60.6) | 27 (11.5) | 207 (88.5) | |
| Female | 152 (39.4) | 11 (7.2) | 141 (92.8) | |
| **Age group** |  |  |  | |
| ≥60 | 157 (40.7) | 21 (13.4) | 136 (86.6) | |
| <60 | 229 (59.3) | 17 (7.4) | 212 (92.6) | |
| **Cancer type** |  |  |  | |
| Non-small cell lung cancer | 129 (33.4) | 2 (1.6) | 127 (98.4) | |
| Melanoma | 185 (47.9) | 33 (17.8) | 152 (82.2) | |
| Clear cell renal cell carcinoma | 35 (9.1) | 2 (5.7) | 33 (94.3) | |
| Bladder cancer | 27 (7.0) | 1 (4.7) | 26 (96.3) | |
| Head and neck cancer | 10 (2.6) | 0 (0) | 10 (100) | |
| **Drug class** |  |  |  | |
| CTLA-4(mono) | 142 (36.8) | 26 (18.3) | 116 (81.7) | |
| PD-(L)1(mono) | 115 (29.8) | 4 (3.1) | 111 (96.9) | |
| CTLA-4 + PD-(L)1 (combo) | 129 (33.4) | 8 (6.2) | 121 (93.8) | |
| **Best overall response** |  |  |  | |
| CR/PR | 118 (30.6) | 20 (16.9) | 98 (83.1) | |
| SD | 94 (24.4) | 9 (9.5) | 85 (90.5) | |
| PD | 163 (42.2) | 9 (5.5) | 154 (94.5) | |
| NE^b^ | 11 (2.8) | 0 (0) | 11 (100) | |
| **Durable clinical benefit** |  |  |  | |
| DCB | 163 (42.2) | 26 (16.0) | 137 (84.0) | |
| NDB | 195 (50.5) | 11 (5.6) | 184 (94.4) | |
| NE^c^ | 28 (7.3) | 1 (3.6) | 27 (96.4) | |
| **EPHA7 status** |  |  |  | |
| EPHA7-WT | 348 (90.2) | 0 (0) | 348 (100) | |
| EPHA7-MUT | 38 (9.8) | 38 (100) | 0 (0) | |
| **Overall patients** | 386 | 38 (9.8) | 348(90.2) | |
| ^a^ Indicated percentage of EPHA7-MUT or EPHA7-WT patients in a given category (i.e. specific gender, specific age group)  ^b^ Eleven patients with best overall response not evaluable due to missing data, including four from Miao et al. 2018 and seven from Hellmann et al. 2018.  ^c^ Twenty-eight patients with durable clinical benefit not evaluable, including 11 missing data and 17 patients who had not progressed but were censored before 6 months of follow-up.  Abbreviations: CR, complete response; CTLA-4, cytotoxic T-cell lymphocyte-4; DCB, durable clinical benefit; NDB, no durable benefit; NE, not evaluable; PD, progressive disease; PD-(L)1 programmed cell death-1 or programmed death-ligand 1; PR, partial response; SD, stable disease. | | | | |

­
