# Supplementary figures and images for: EPHA7 mutation as a predictive biomarker for immune checkpoint inhibitors in multiple cancers
Source: BMC Med. 2021 Feb 2;19:26. doi: 10.1186/s12916-020-01899-x (PMC7852135; doi:10.1186/s12916-020-01899-x)

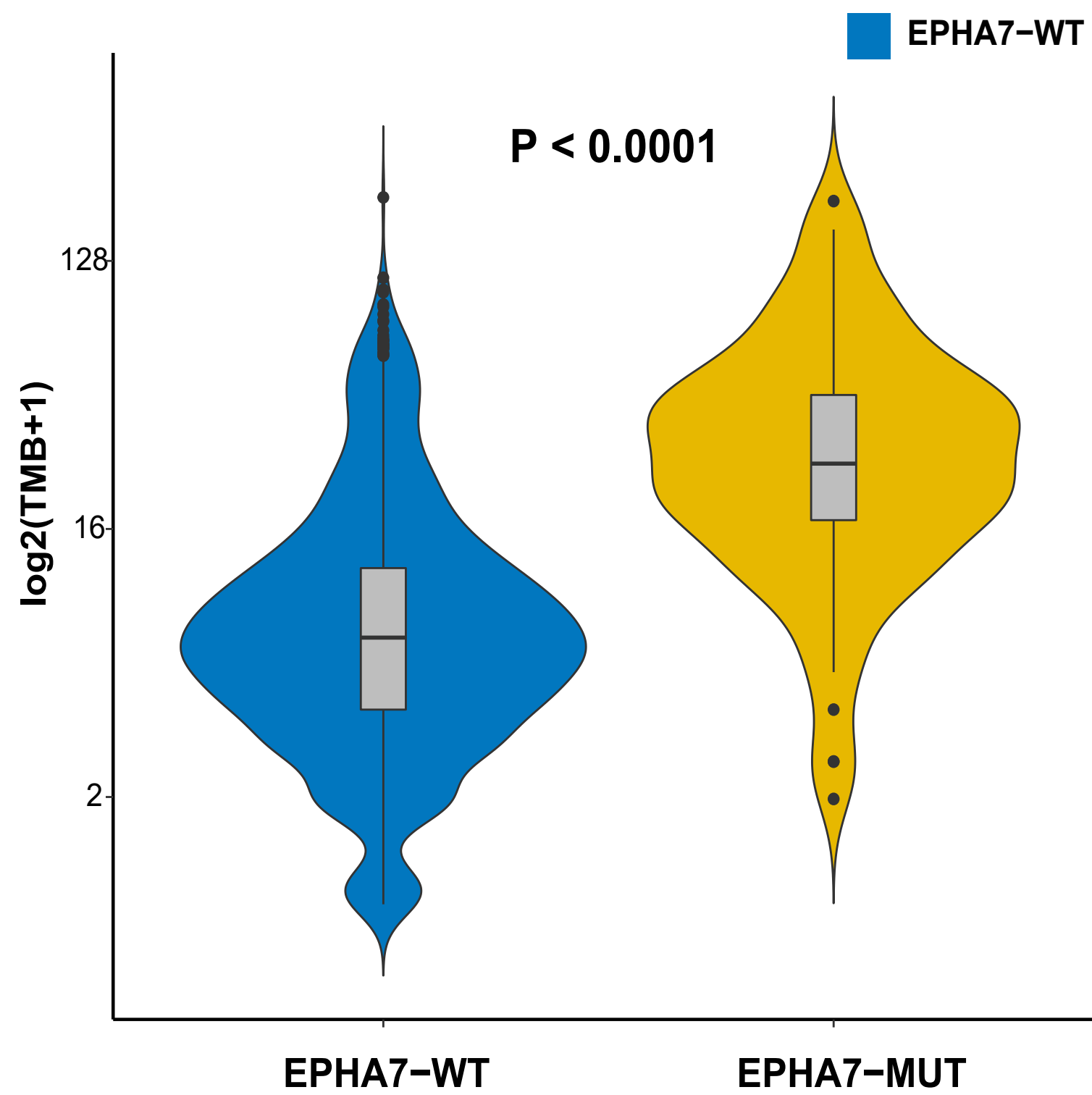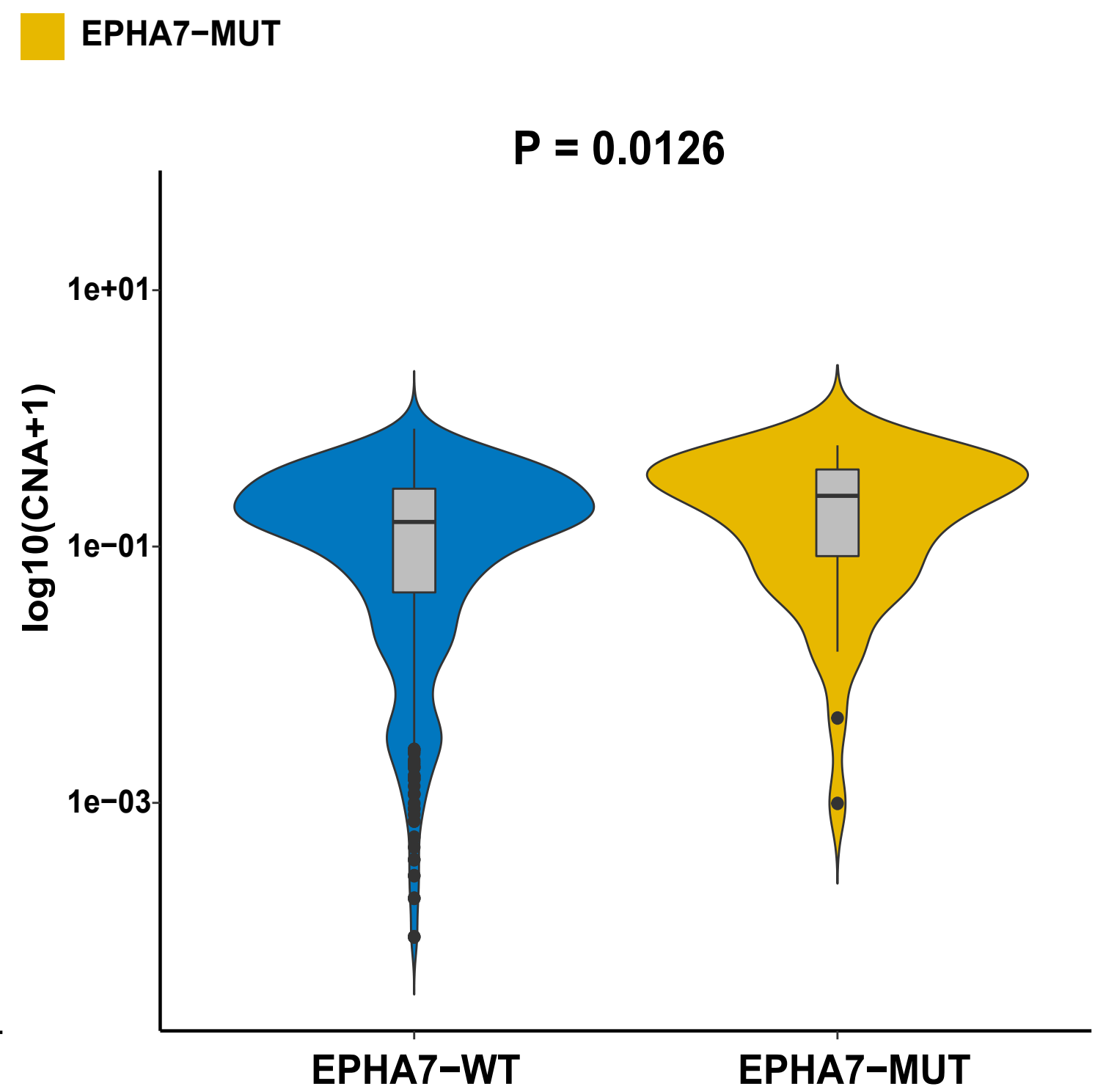

Supplement: Supplementary file 3 — Additional file 3: Figure S1. Violin plot depicting the distribution of TMB and CNA in EPHA7-MUT and EPHA7-WT tumors. [file 12916_2020_1899_MOESM3_ESM.pdf]

**A****Discovery cohort**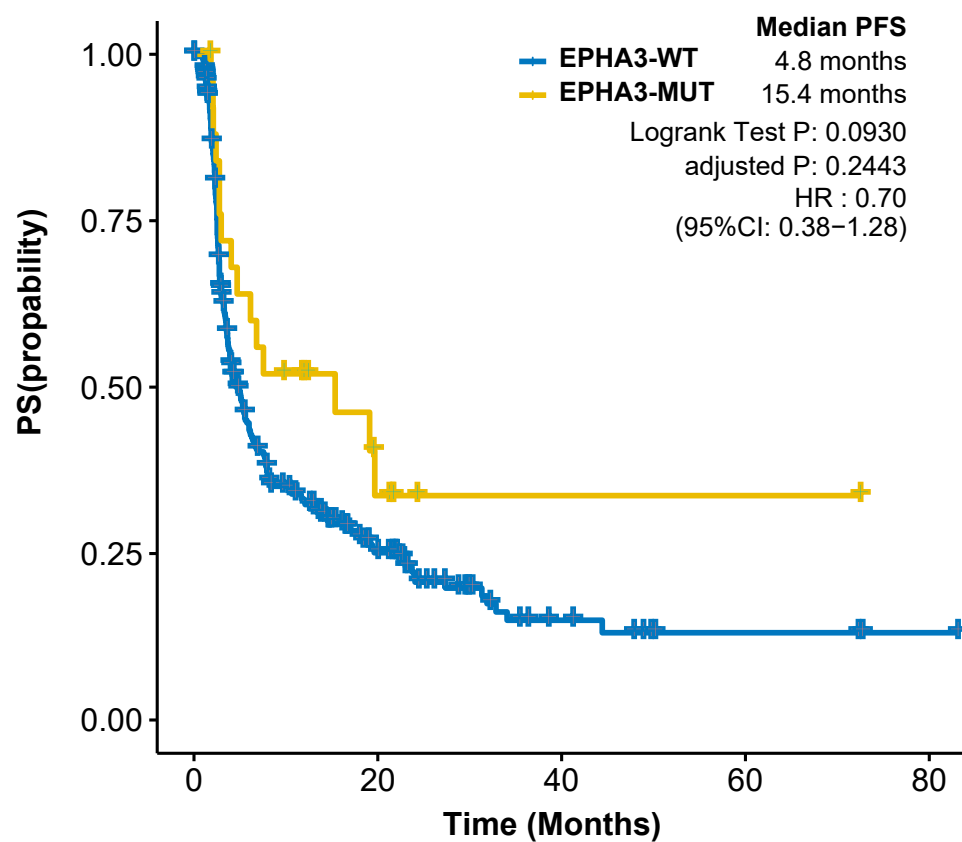**B****Discovery cohort**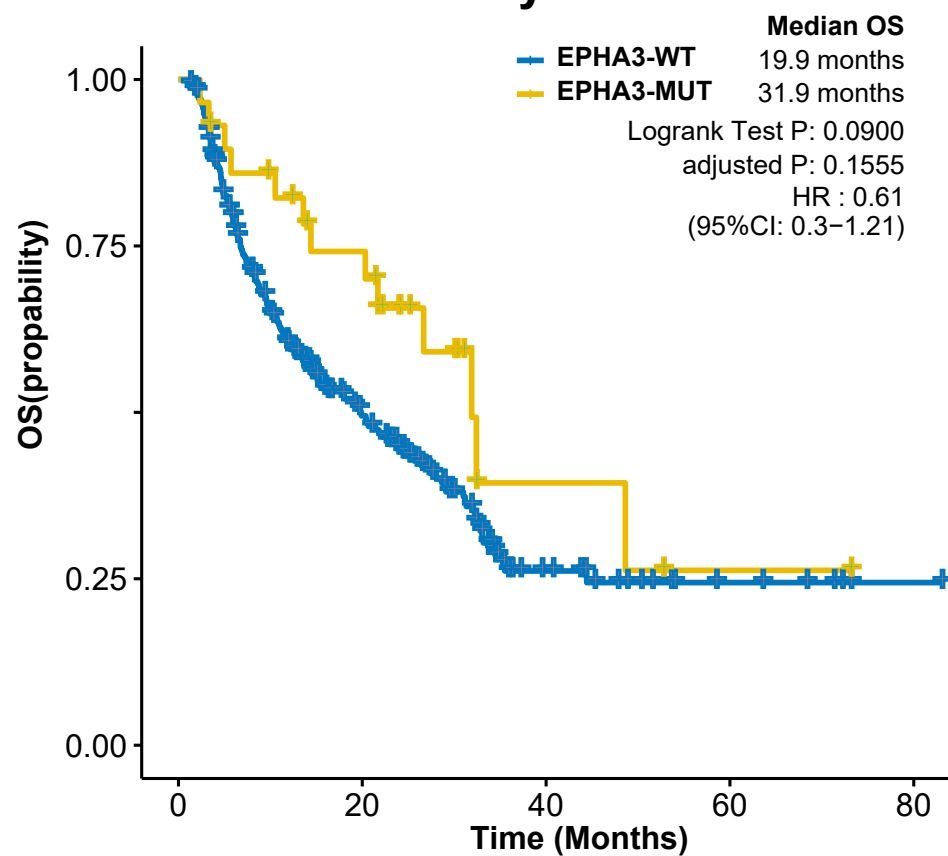**C****Validation cohort**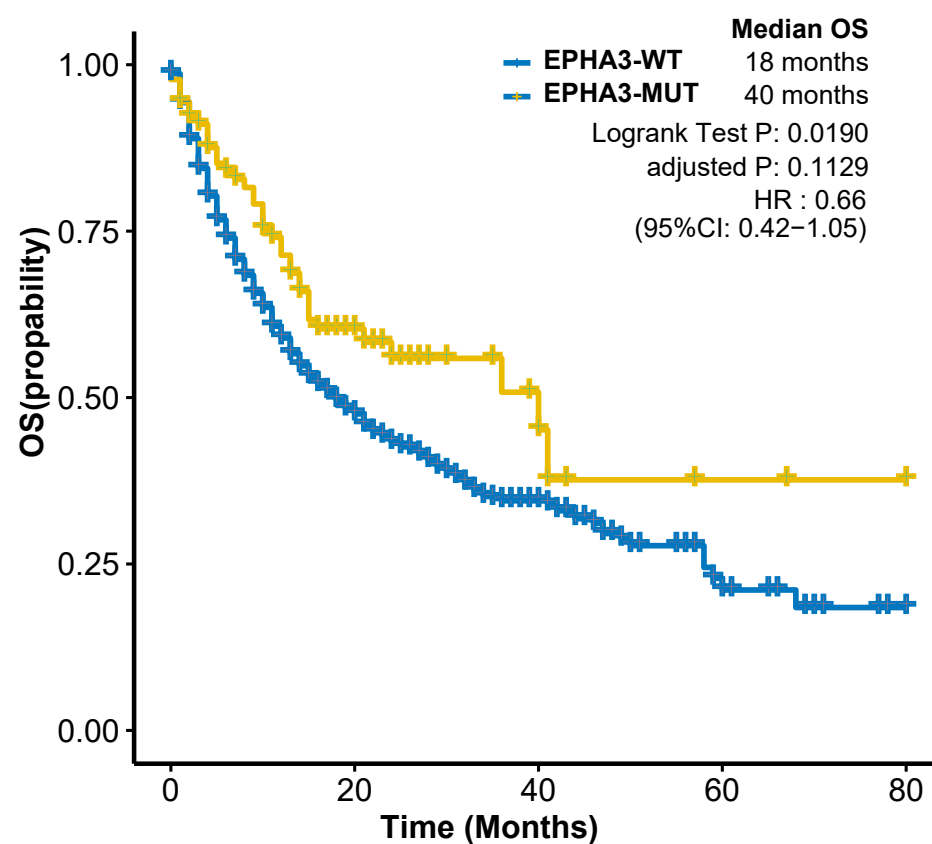

Supplement: Supplementary file 8 — Additional file 8: Figure S4. Survival analysis of EPHA3 in both discovery and validation cohort. [file 12916_2020_1899_MOESM8_ESM.pdf]

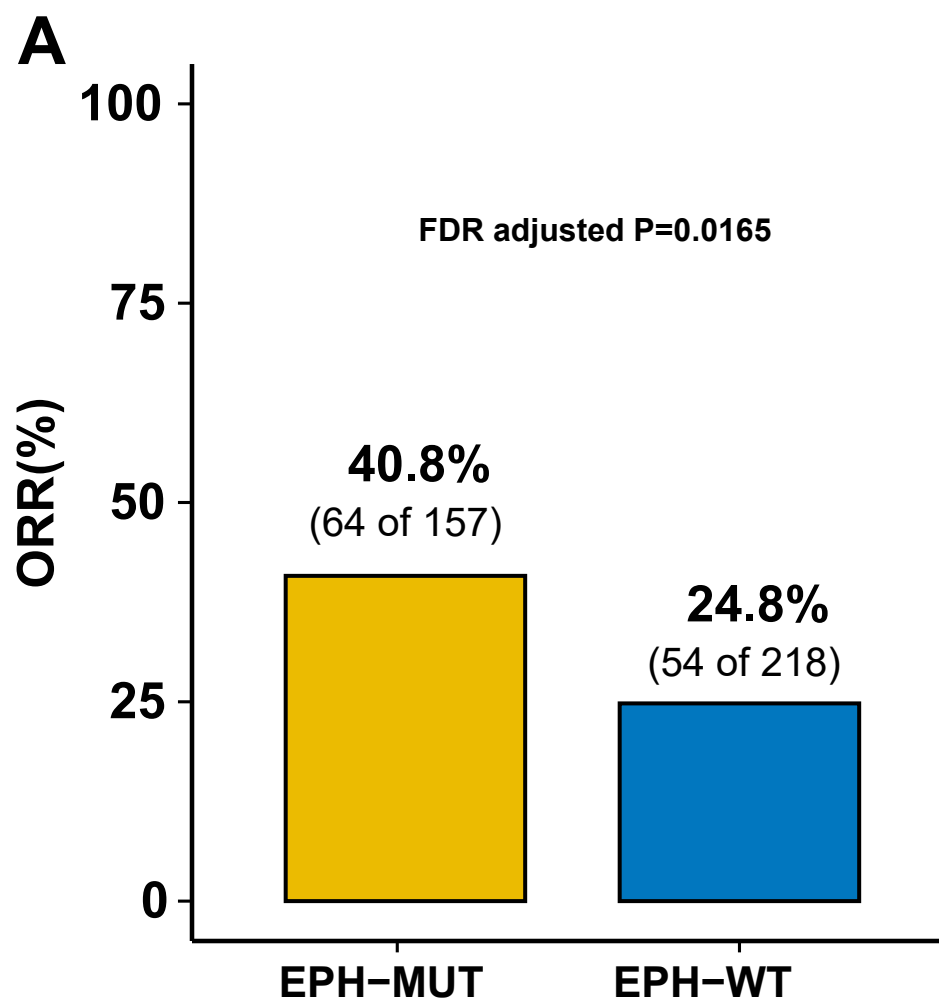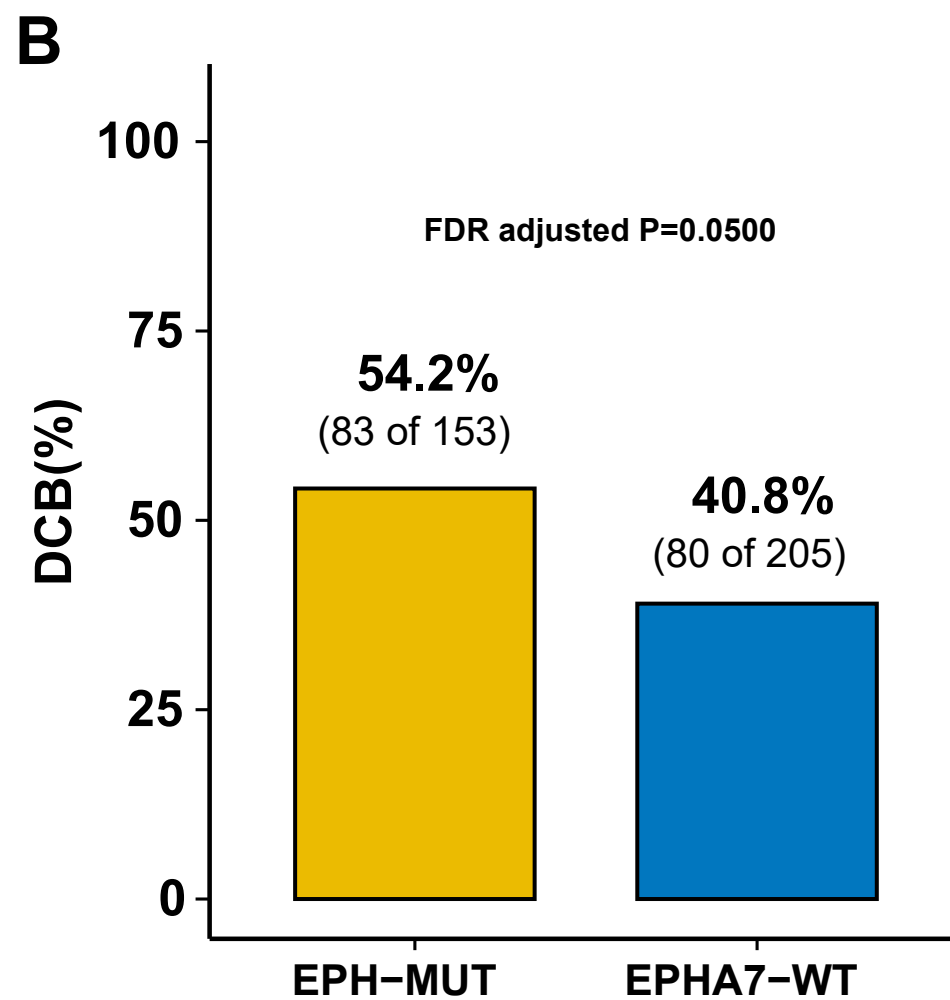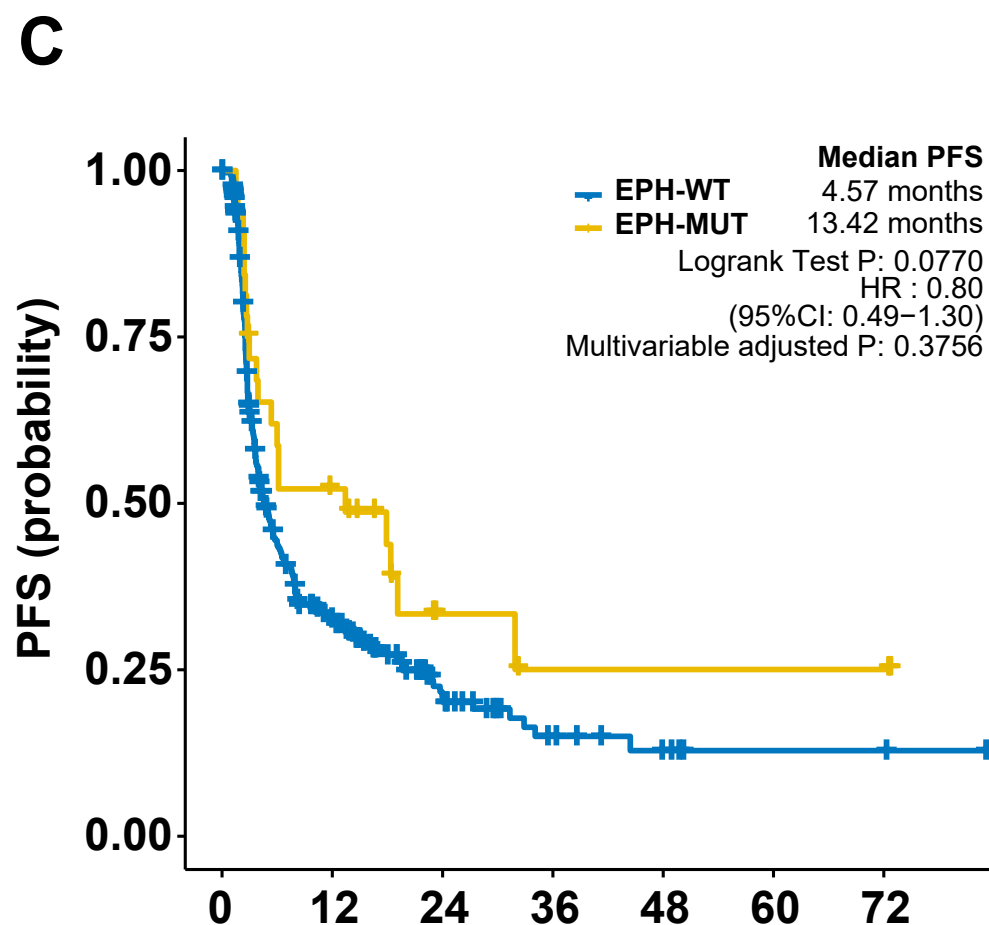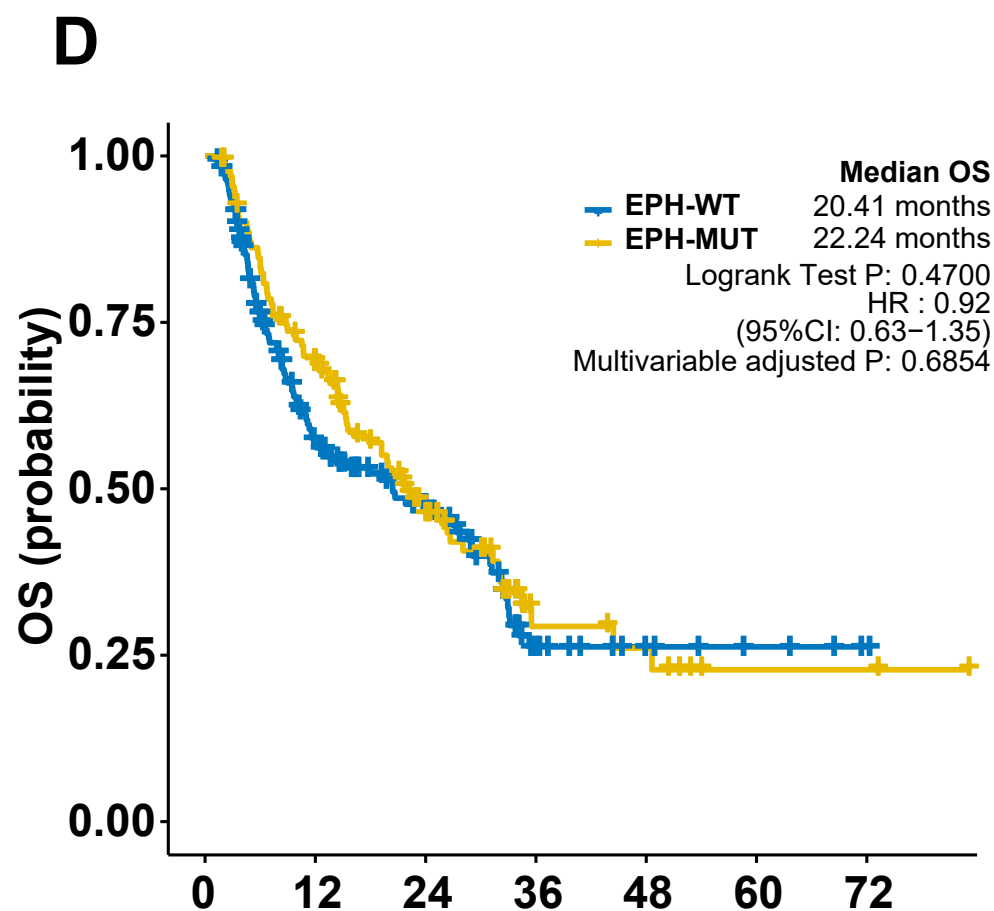

Supplement: Supplementary file 9 — Additional file 9: Figure S5. Association between clinical outcomes and the combination of all 14 EPH genes in the discovery cohort. [file 12916_2020_1899_MOESM9_ESM.pdf]
