# Supplementary material for: EPHA7 mutation as a predictive biomarker for immune checkpoint inhibitors in multiple cancers
Source: BMC Med. 2021 Feb 2;19:26. doi: 10.1186/s12916-020-01899-x (PMC7852135; doi:10.1186/s12916-020-01899-x)
Supplement: Supplementary file 4 — Additional file 4: Figure S2. Truncating vs non-truncating EPHA7 mutation analysis in both discovery and validation cohort. Figure S2 A-D: discovery cohort. Figure S2 E: validation cohort. [file 12916_2020_1899_MOESM4_ESM.pdf]

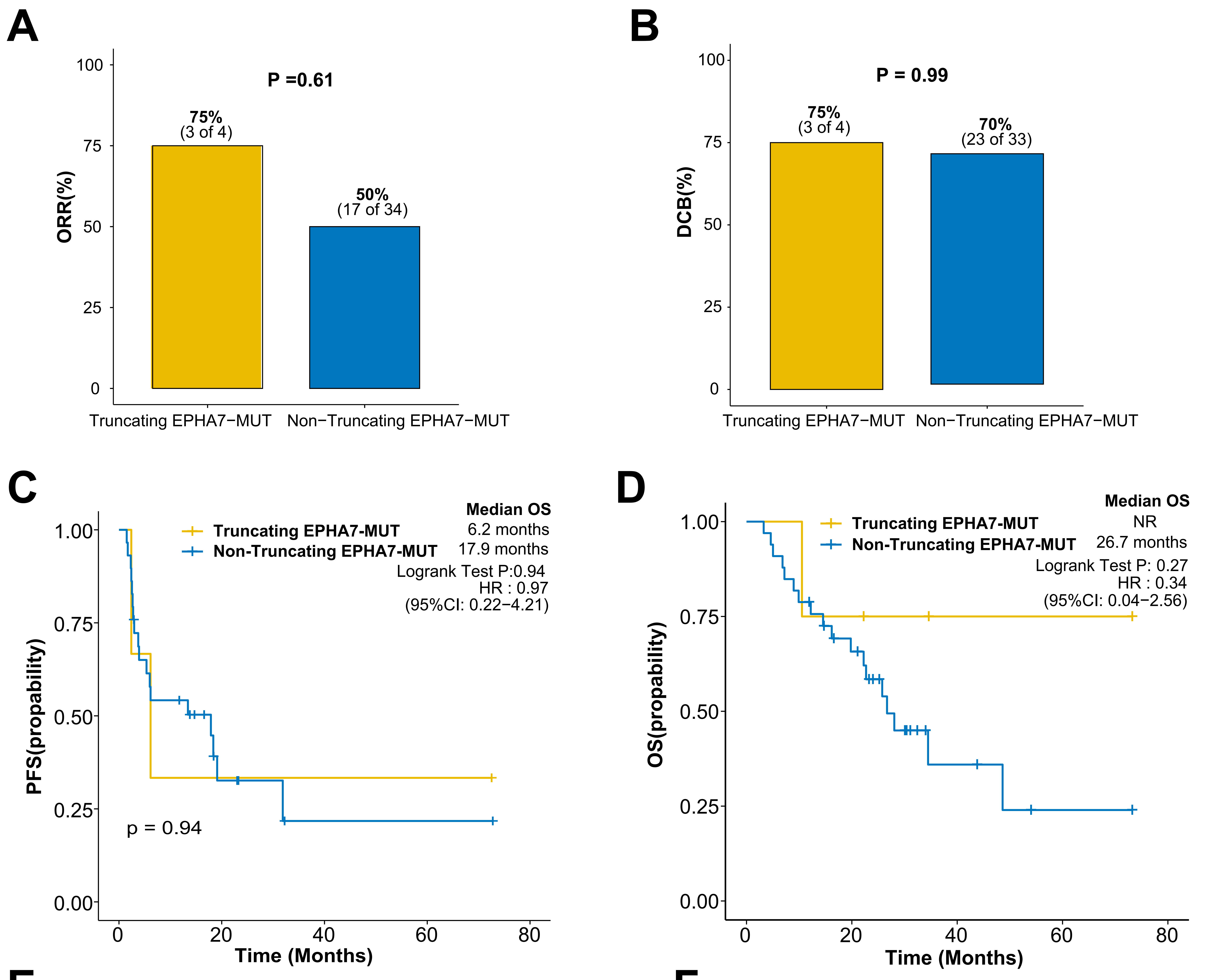

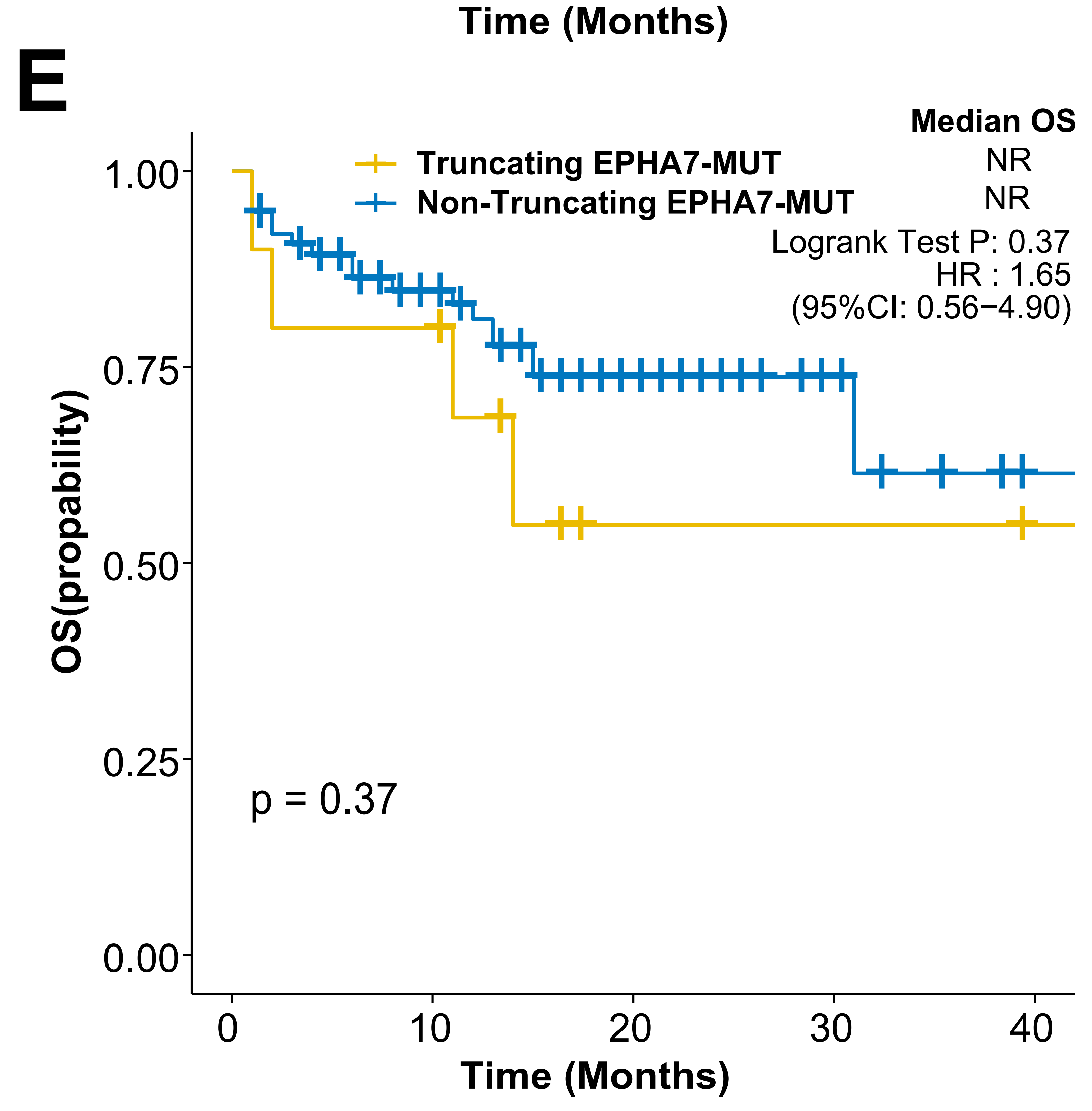

F

No. of patients in Discovery cohort	
Truncating EPHA7-MUT	NonTruncating EPHA7-MUT
4	34
**No. of patients in Validation cohort**	
Truncating EPHA7-MUT	NonTruncating EPHA7-MUT
10	73
