# Supplementary material for: EPHA7 mutation as a predictive biomarker for immune checkpoint inhibitors in multiple cancers
Source: BMC Med. 2021 Feb 2;19:26. doi: 10.1186/s12916-020-01899-x (PMC7852135; doi:10.1186/s12916-020-01899-x)
Supplement: Supplementary file 7 — Additional file 7: Figure S3. Survival analysis of cancer subgroups in the TCGA cohort. [file 12916_2020_1899_MOESM7_ESM.pdf]

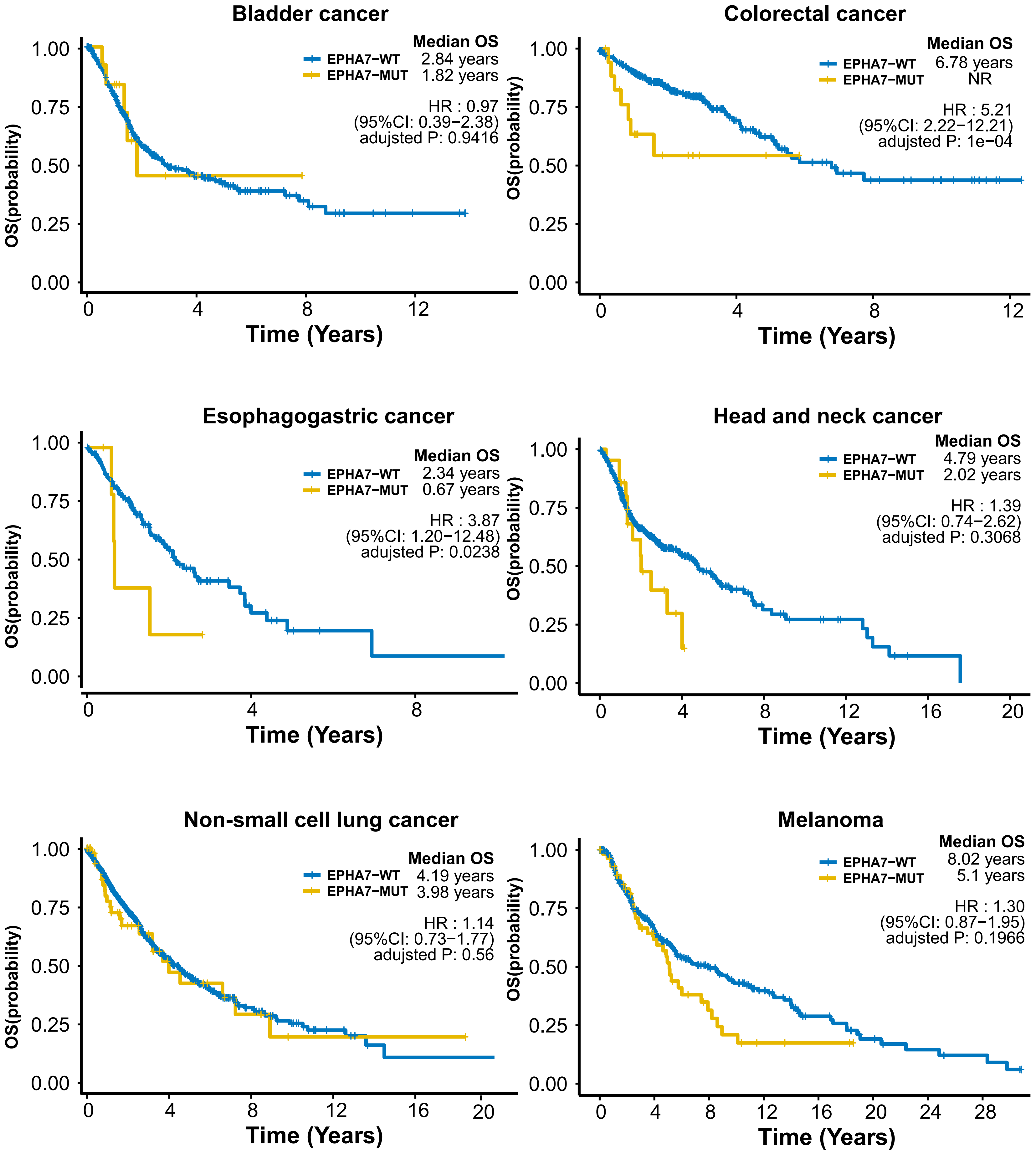
Bladder cancer
Median OS
EPHA7−WT 2.84 years
EPHA7−MUT 1.82 years
HR : 0.97
(95%CI: 0.39−2.38)
adujsted P: 0.9416
OS(probability)
1.00
0.75
0.50
0.25
0.00
0
4
8
12
Time (Years)
Colorectal cancer
Median OS
EPHA7−WT 6.78 years
EPHA7−MUT NR
HR : 5.21
(95%CI: 2.22−12.21)
adujsted P: 1e−04
OS(probability)
1.00
0.75
0.50
0.25
0.00
0
4
8
12
Time (Years)
Esophagogastric cancer
Median OS
EPHA7−WT 2.34 years
EPHA7−MUT 0.67 years
HR : 3.87
(95%CI: 1.20−12.48)
adujsted P: 0.0238
OS(probability)
1.00
0.75
0.50
0.25
0.00
0
4
8
Time (Years)
Head and neck cancer
Median OS
EPHA7−WT 4.79 years
EPHA7−MUT 2.02 years
HR : 1.39
(95%CI: 0.74−2.62)
adujsted P: 0.3068
OS(probability)
1.00
0.75
0.50
0.25
0.00
0
4
8
12
16
20
Time (Years)
Non-small cell lung cancer
Median OS
EPHA7−WT 4.19 years
EPHA7−MUT 3.98 years
HR : 1.14
(95%CI: 0.73−1.77)
adujsted P: 0.56
OS(probability)
1.00
0.75
0.50
0.25
0.00
0
4
8
12
16
20
Time (Years)
Melanoma
Median OS
EPHA7−WT 8.02 years
EPHA7−MUT 5.1 years
HR : 1.30
(95%CI: 0.87−1.95)
adujsted P: 0.1966
OS(probability)
1.00
0.75
0.50
0.25
0.00
0
4
8
12
16
20
24
28
Time (Years)
